# Role of neuroinflammation in the emotional and cognitive alterations displayed by animal models of obesity

**DOI:** 10.3389/fnins.2015.00229

**Published:** 2015-07-03

**Authors:** Nathalie Castanon, Giamal Luheshi, Sophie Layé

**Affiliations:** ^1^Nutrition and Integrative Neurobiology, INRA, UMR 1286, Université de BordeauxBordeaux, France; ^2^Department of Psychiatry, Douglas Mental Health University Institute, McGill UniversityMontreal, Canada

**Keywords:** obesity, anxiety, depression, memory impairments, inflammation, neuroinflammation, indoleamine 2-3-dioxygenase, GTP-cyclohydrolase 1

## Abstract

Obesity is associated with a high prevalence of mood disorders and cognitive dysfunctions in addition to being a significant risk factor for important health complications such as cardiovascular diseases and type 2 diabetes. Identifying the pathophysiological mechanisms underlying these health issues is a major public health challenge. Based on recent findings, from studies conducted on animal models of obesity, it has been proposed that inflammatory processes may participate in both the peripheral and brain disorders associated with the obesity condition including the development of emotional and cognitive alterations. This is supported by the fact that obesity is characterized by peripheral low-grade inflammation, originating from increased adipose tissue mass and/or dysbiosis (changes in gut microbiota environment), both of which contribute to increased susceptibility to immune-mediated diseases. In this review, we provide converging evidence showing that obesity is associated with exacerbated neuroinflammation leading to dysfunction in vulnerable brain regions associated with mood regulation, learning, and memory such as the hippocampus. These findings give new insights to the pathophysiological mechanisms contributing to the development of brain disorders in the context of obesity and provide valuable data for introducing new therapeutic strategies for the treatment of neuropsychiatric complications often reported in obese patients.

## Introduction

The prevalence of obesity has been steadily and alarmingly increasing worldwide for decades, fostering a rise in serious obesity-related outcomes, particularly cardiovascular diseases and metabolic disorders that contribute to a significant rise in mortality. In addition, obesity is also increasingly linked to a number of psychopathologies including mood disorders and cognitive dysfunctions (Luppino et al., 2010; Francis and Stevenson, 2013). The incidence of depressive symptoms (up to 30%) is much higher in obese subjects than in normal weight age-matched population (Roberts et al., 2010; Pan et al., 2012; Lin et al., 2013). Similarly, convergent clinical studies have revealed a predictive longitudinal association between obesity and the development of age-related cognitive deficits (Cournot et al., 2006; Sabia et al., 2009; Dahl et al., 2013). Obesity is also a risk factor for neurological diseases such as Alzheimer's disease (Frisardi et al., 2010; Farooqui et al., 2012). Interestingly, significant improvement in mood and cognitive function is reported after weight loss induced by bariatric surgery or diet restriction in obese subjects (Brinkworth et al., 2009; Andersen et al., 2010; Siervo et al., 2012; Alosco et al., 2014). Neuropsychiatric comorbidities considerably impair the quality of life and social functioning of obese individuals. More importantly, they emerge as additional risk factors for aggravation of obesity and related systemic pathological complications (Fiedorowicz et al., 2008; Scott et al., 2008). Indeed, lifetime history of neuropsychiatric disorders or stressful life events are reliable predictors for weight gain and the subsequent development of obesity (Goldbacher et al., 2009; Luppino et al., 2010; McIntyre et al., 2012). In addition, weight gain often appears as a common side effect of many psychiatric medications (Lopresti and Drummond, 2013). Altogether, these data point to a noxious bidirectional relationship between obesity and neuropsychiatric disorders. Identifying the pathophysiological mechanisms that underlie such a comorbid association represents therefore a major public health challenge.

Diet, social factors, and psychological distress are classically advanced as contributors to the development of neuropsychiatric symptoms in obese individuals. Mechanistically, these factors may impact the functioning of key biological systems participating in both obesity and neuropsychiatric disorders and able to target the central nervous system (CNS) (McIntyre et al., 2012; Hryhorczuk et al., 2013; Lopresti and Drummond, 2013). Although several biological components of obesity are likely candidates, there is increasing evidence for a role of inflammation in this process (Emery et al., 2007; Capuron et al., 2008, 2011a; Castanon et al., 2014; Lasselin and Capuron, 2014). Severe obesity is indeed associated with an inflammatory profile characterized by increased concentrations of circulating cytokines (Cancello and Clement, 2006). The effect of inflammation on brain function and behavior has been reported in many other chronic inflammatory conditions (Evans et al., 2005; Dantzer et al., 2008). However, there remains a noticeable void in understanding the pathophysiological mechanisms underlying the development of neuropsychiatric symptoms in the context of obesity. We describe here how animal models of obesity, which already shed light on the mechanisms of weight control and associated metabolic alterations, can be useful to address this issue. We also review the available evidence showing that obesity is associated with exacerbated neuroinflammation leading to dysfunction in vulnerable brain regions associated with mood regulation, learning and memory, especially the hippocampus.

## Obesity: more than just a metabolic disorder

Obesity is defined by the *World Health Organization* as an “excessive or abnormal fat accumulation that presents a risk to health.” Weight gain is associated with marked hyperplasia of the white adipose tissue and substantial changes in the function of adipocytes that start to secrete a number of bioactive molecules collectively referred to as adipokines (leptin, adiponectin, and a multitude of pro- and anti-inflammatory cytokines) (Lehr et al., 2012; Aguilar-Valles et al., 2015). Due to their ability of acting remotely on different organs these molecules are suspected to participate in the etiology of many obesity-associated metabolic comorbidities (Lehr et al., 2012; Aguilar-Valles et al., 2015; Bluher and Mantzoros, 2015). Chronic obesity is indeed often associated with hypertension, coronary artery disease, dyslipidemia, hyperleptinemia, and impaired glucose tolerance linked to hyperinsulinemia and insulin resistance. Along with metabolic dysregulations, basal low-grade inflammation increasingly appears as another key component of obesity, which is now considered not only as a metabolic disorder but also as an inflammatory condition affecting both the innate and acquired immune systems (Schmidt and Duncan, 2003; Cancello and Clement, 2006; Gregor and Hotamisligil, 2011). Elevated plasma levels of inflammatory cytokines (including interleukin (IL)-1β, tumor necrosis factor (TNF)-α, IL-6 and C-reactive protein) have been reported in obese patients (Park et al., 2005; Capuron et al., 2011a) and different animal models of obesity (Xu et al., 2003; De Souza et al., 2005; Cani et al., 2009; Pistell et al., 2010; Dinel et al., 2011, 2014; Lawrence et al., 2012). Interestingly, peripheral low-grade inflammation is significantly improved following weight loss induced by low-caloric diet or bariatric surgery in both obese humans (Ryan and Nicklas, 2004; Manco et al., 2007; Belza et al., 2009; Hakeam et al., 2009; Rao, 2012) and animals (Zhang et al., 2011; Liu et al., 2014; Schneck et al., 2014). Obesity is also characterized by increased susceptibility to immune-mediated diseases (Kanneganti and Dixit, 2012) and to infections (Amar et al., 2007; Rummel et al., 2010; Lawrence et al., 2012; Huttunen and Syrjanen, 2013; Dinel et al., 2014).

## Obesity: an inflammatory condition

One major player in the development of the chronic low-grade inflammatory state characterizing obesity is the white adipose tissue, consistent with findings showing associations between circulating levels of cytokines and measures of central adiposity (Park et al., 2005). Together with adipocytes, infiltrated macrophages, and T cells that progressively accumulate in the adipose tissue potently secrete inflammatory mediators (Cancello and Clement, 2006; Gregor and Hotamisligil, 2011; Zeyda et al., 2011; Lasselin et al., 2013; Kim et al., 2014). In addition, accumulation of activated immune cells also releasing inflammatory factors in the circulation has been reported within other organs, in particular the liver and muscles (Pedersen and Febbraio, 2012; McNelis and Olefsky, 2014). More recently, gut microbiota alterations and increased gut permeability have also been involved in the pathogenesis of obesity and related metabolic comorbidities (Brun et al., 2007; Tehrani et al., 2012; Finelli et al., 2014), in particular through their impact on local and systemic inflammation (Cani et al., 2009, 2012; Verdam et al., 2013). Specific microbes-associated molecular patterns (MAMPs) such as gut microbiota-derived lipopolysaccharide (LPS) have been indeed shown to play a major role in the onset and progression of obesity-related inflammation and metabolic diseases (Cani et al., 2007; Creely et al., 2007). Chronic intake of high-fat diet impairs gut permeability, which leads to the instauration of metabolic endotoxemia (i.e., increased plasma levels of LPS) contributing to obesity-related inflammation by activating systemic macrophages (Cani et al., 2008, 2009; Verdam et al., 2013). Conversely, reduced serum levels of an endotoxemia marker, the LPS binding protein, are found in obese individuals following weight loss (Cani et al., 2008; Yang et al., 2014).

Whatever the mechanisms triggering systemic inflammation in obesity, it is now clear that this inflammatory state contributes to increased central inflammatory processes (Rummel et al., 2010; Buckman et al., 2014) associated with both metabolic dysregulations and behavioral alterations (Dantzer et al., 2008). Obesity-related brain inflammation is particularly notable in the hypothalamus, with local enhanced inflammatory cytokine expression and activation of dependent signaling pathways being repeatedly reported in diet induced obesity (DIO) models (De Souza et al., 2005; Zhang et al., 2008; Kleinridders et al., 2009; Cai and Liu, 2012; Gao et al., 2014; Maric et al., 2014). Increased infiltration, activation, and proliferation of microglia and astrocytes in the hypothalamus are also noted in both obese humans and rodent models of obesity (Thaler et al., 2012). Interestingly, increased hypothalamic inflammation has been related to the metabolic dysregulations characterizing severe obesity, including leptin resistance, insulin resistance and hyperglycemia (De Souza et al., 2005; Velloso et al., 2008; Zhang et al., 2008; Kleinridders et al., 2009). Beyond basal hypothalamic inflammation that appears as a function of obesity, we also demonstrated in DIO models a marked exacerbation of hypothalamic expression of different inflammatory factors following a systemic immune challenge, namely acute intraperitoneal injection of LPS (Pohl et al., 2009; André et al., 2014). Of note, systemic LPS challenge also exacerbates expression of brain cytokines (IL-1β, TNF-α) in areas involved in mood regulation and memory formation such as the hippocampus (André et al., 2014; Boitard et al., 2014; Dinel et al., 2014). Besides, significant hippocampal inflammation is also reported in unstimulated conditions when more severe obesity is modeled (Dinel et al., 2011, 2014; Erion et al., 2014). For example, immune defects such as increased systemic inflammation and/or reduced immune competence (Cani et al., 2009; Rummel et al., 2010; Lawrence et al., 2012), which are reported in genetic models of severe obesity [*ob/ob* (deficient for leptin) and *db/db* (deficient for functional leptin receptor) mice], are associated with increased hippocampal cytokine (IL-1β, IL-6, TNF-α) expression (Dinel et al., 2011, 2014; Erion et al., 2014). Importantly, hippocampal inflammation is associated with signs of dysfunctions within the brain (Stranahan et al., 2008; Dey et al., 2014; Erion et al., 2014) and marked emotional and cognitive alterations (Stranahan et al., 2008; Dinel et al., 2011, 2014; Erion et al., 2014). These models of obesity are therefore especially suited for studying the long-term adverse effects of obesity and investigating in particular the molecular and cellular events underlying the development of emotional and cognitive alterations.

## Cognitive and emotional alterations displayed by animal models of obesity

Several complementary models of obesity have been developed over the last decades in order to study obesity and related health complications, particularly cardiovascular diseases or type 2 diabetes (Varga et al., 2009; Kanasaki and Koya, 2011). Beyond these research topics, such animal models proved to be very useful for studying the neurobiological basis of obesity-associated emotional and cognitive alterations (Biessels and Gispen, 2005; Kanoski and Davidson, 2011). For example, models relying on diet modifications (DIO models), which are close to human obesity with respect to etiological aspects, give the opportunity of controlling the degree of obesity (low to moderate), as well as the duration and time of exposure to high-fat diet (perinatal periods, childhood, adulthood). Moreover, because of their longitudinal characteristic, DIO models allow for investigating the pathophysiological changes preceding the development of obesity-related comorbidities, including neuropsychiatric alterations. In addition, genetic models of obesity reproduce moderate to severe obesity and display most of the metabolic, inflammatory, and brain alterations characterizing this condition, including neurobehavioral alterations.

In agreement with clinical studies, experimentally-induced obesity is associated with a wide array of cognitive abnormalities including impairment in learning and memory (Kanoski and Davidson, 2011). For example, performances of spatial learning and long-term memory (Boitard et al., 2012, 2014; Valladolid-Acebes et al., 2013; André et al., 2014) or contextual fear conditioning (Hwang et al., 2010) are impaired in DIO models together with hippocampal synaptic plasticity (Molteni et al., 2002; Hwang et al., 2010; Boitard et al., 2012). Interestingly, the juvenile period (from weaning to young adulthood) seems to be particularly vulnerable to the adverse effects of high-fat diet on hippocampal function and related learning and memory processes (Valladolid-Acebes et al., 2013; Boitard et al., 2014). Indeed, exposure of adult mice to high-fat diet for the same duration as young mice does not yield such behavioral nor hippocampal alterations (Boitard et al., 2014; Valladolid-Acebes et al., 2013). Moreover, we recently showed that rats exposed to high-fat diet during the perinatal period and maintained on this diet during adulthood displayed impaired spatial memory and hippocampal neurogenesis in contrast with those exposed to the diet only during the perinatal period or after weaning (Lepinay et al., 2015). These data suggest therefore that exposure to high-fat diet during the perinatal period increases hippocampal vulnerability to the adverse effects of subsequent high-fat feeding. This study complements previously published data showing that maternal consumption of a high-fat diet can affect spatial memory (Bilbo and Tsang, 2010; Tozuka et al., 2010; Page et al., 2014) and hippocampal function in off-spring (Niculescu and Lupu, 2009; Tozuka et al., 2009). Similarly, an association between impaired hippocampus-dependent spatial memory performances in the water-maze or Y-maze tasks (Li et al., 2002; Dinel et al., 2011) and altered hippocampal neurogenesis, neuronal dendrite morphology, and synaptic plasticity is also found in genetic models of obesity such as *db/db* mice (Stranahan et al., 2008, 2009; Erion et al., 2014; Ramos-Rodriguez et al., 2014). Interestingly, these mice also display increased hippocampal expression of cytokines that are known to influence hippocampal plasticity and behavior (Dinel et al., 2011). In addition, blockade of hippocampal IL-1β expression in *db/db* mice normalizes hippocampal dendritic spine density and prevents synaptic dysfunction and cognitive impairment (Erion et al., 2014). On the other hand, acquisition of a conditioned taste aversion learning task (Ohta et al., 2003), as well as working memory performances tested in a hippocampus-independent task are on the contrary preserved in *db/db* mice (Dinel et al., 2011). Altogether, these data point to the importance of the hippocampus as key brain area for mediating cognitive impairments linked to obesity.

Emotional reactivity is also impaired in animal models of obesity, although with a different time-course of development than cognition. Interestingly, we recently showed that DIO starting at weaning in mice alters first spatial memory, then anxiety-like behavior, whereas depressive-like behavior, either assessed in the forced swim test (FST) or tail suspension test (TST), remains unchanged unless the animals are challenged with LPS (André et al., 2014). Rats exposed to moderate high-fat diet from the perinatal period and throughout life also display memory impairments but unaltered depressive-like behavior in unstimulated conditions (Lepinay et al., 2015). Of note, increased basal depressive-like behavior has been previously reported in other DIO models but only when very high-fat diets associated with important metabolic dysfunctions and presumably basal low-grade inflammation are used (Yamada et al., 2011; Sharma and Fulton, 2013). Moreover, it is well-known that depressive-like behaviors mostly increase under challenging conditions such as stress exposure (Lu et al., 2008) or immune stimulation (Frenois et al., 2007; Moreau et al., 2008). Similarly, depressive-like behavior is also increased in obese mice compared to lean controls when experimental conditions used are particularly stressful (e.g., sustained and/or repeated exposure to the test, successive exposures to different behavioral tests over short periods of time) (Collin et al., 2000; Yamada et al., 2011). However, when stressful factors are tightly controlled, *db/db* mice display similar levels of depressive-like behavior than their lean *db/+* controls, although anxiety-like behavior remains elevated (Dinel et al., 2011). These results strongly suggest an important role for the inflammatory system and/or the hypothalamo-pituitary-adrenal axis (HPA) axis, which are functionally related (Raison and Miller, 2003), in underlying emotional alterations associated with obesity. In addition, they indicate that obesity, in conjunction with environmental conditions, can amplify CNS dysfunctions and/or their harmful consequences on mood and cognition. Supporting this notion, it has been recently shown in DIO mice that exacerbated neuroinflammatory response to a systemic LPS challenge contributes to increase depressive-like behavior (Aguilar-Valles et al., 2014). Moreover, exacerbated neuroinflammation and depressive-like behavior displayed by LPS-injected DIO mice is also accompanied by enhanced HPA axis activation (André et al., 2014). Altogether, these data support the notion that obesity-associated cognitive and emotional alterations rely on interactions involving multiple systems, including metabolic characteristics, environmental influences and immune-related processes.

## Brain inflammation: a key player in the control of cognitive functions and mood

### From sickness behavior to neuropsychiatric symptoms

Over the last decades, clinical (Evans et al., 2005; Raison et al., 2010; Capuron and Miller, 2011; Lasselin and Capuron, 2014) and experimental (Castanon et al., 2002; Frenois et al., 2007; Moreau et al., 2008) investigations focusing on the intricate relationship between the innate immune system and the brain have supported a main role for dysregulated production and/or brain action of cytokines in neuropsychiatric disorders through the profound action they exert on brain functions (Dantzer et al., 2008; Zunszain et al., 2012). Most cytokines have little or no function in healthy tissues, but they are rapidly induced locally by activated innate immune cells in response to tissue injury, infection, or inflammation. They are then able to act systemically on distant organs, including the brain, through a number of non-exclusive humoral, neural, and cellular pathways that allow the peripheral immune messages to be transmitted to the brain (Dantzer et al., 2008; Capuron and Miller, 2011). Activation of immune-to-brain communication ultimately induces the production of brain cytokines by activated endothelial and glial cells, particularly microglia (Layé et al., 1994; Castanon et al., 2004). Upon detection of homeostatic disturbances, microglia are transiently activated and rapidly engaged in brain adaptive immune responses mainly due to their ability to produce cytokines, express their receptors and amplify their signals (Ransohoff and Perry, 2009; Kettenmann et al., 2011). Within the brain, cytokines are able to influence pathways involved in behavioral regulations, including neurotransmitter metabolism and function, neuroendocrine activity, neural plasticity, and/or brain circuitry (Dantzer et al., 2008). During an infection, transient brain cytokine activation thus coordinate a large number of behavioral changes (including weakness, listlessness, malaise, anorexia, fatigue, and transient cognition and mood alterations) collectively referred to as sickness behavior. Necessary for infection recovery, sickness behavior usually resolves within few days, once microbial pathogens have been cleared and the innate immune system is no longer activated. However, failure to tightly regulate systemic immune activation and/or brain microglial activation leads to significant and prolonged induction of peripheral and brain cytokines. This induction in turn might culminate in medical conditions adversely affecting clinical outcomes, including neuropsychiatric symptoms, particularly when they ultimately affect key brain areas, such as the hippocampus, the cortex, or the amygdala (Dantzer et al., 2008). During the last decade, these findings have prompted a surge of interest for the circumstances precipitating the development of such pathological conditions and the identification of the underlying mechanisms.

### Inflammation-associated behavioral alterations: underlying mechanisms

**Figure 1:**
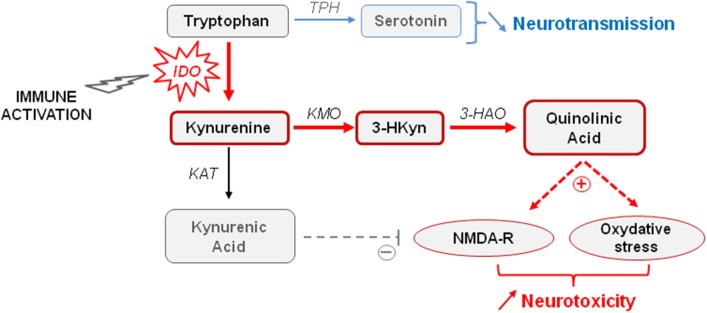
**Tryptophan metabolism through the kynurenine pathway**. Increased indoleamine 2,3-dioxygenase (IDO) activity occurring in activated monocytes, macrophages, and brain microglia in conditions of immune activation catabolizes tryptophan in kynurenine. Tryptophan is the biosynthetic precursor for the synthesis of serotonin. By reducing the availability of tryptophan for serotonin synthesis, IDO activation is able to impair serotonin neurotransmission. Kynurenine is metabolized in different neuroactive glutamatergic metabolites, including 3-hydroxykynurenine (3-HKyn) and quinolinic acid that are produced by activated microglia, and kynurenic acid produced by astrocytes. Elevated levels of quinolinic acid are neurotoxic by activating glutamatergic NMDA receptors and promoting oxidative stress. High concentrations of kynurenic acid can be neuroprotective by antagonizing NMDA receptors, but sustained microglia activation rather promotes increased neurotoxicity. KAT, kynurenine aminotransferase; KMO, kynurenine monooxygenase; 3-HAO, 3-hyrdoxyanthranilic acid oxygenase; TPH, tryptophan hydroxylase.

Converging clinical findings have shown that inflammation-related sickness behavior and neuropsychiatric symptoms differ by their respective duration and intensity, suggesting that they likely have distinct underlying mechanisms (Raison et al., 2010; Capuron and Miller, 2011). Cytokines are able to induce the synthesis of different enzymes in activated monocytes, macrophages and brain microglia, namely the indoleamine 2,3-dioxygenase (IDO) and GTP-cyclohydrolase 1 (GTP-CH1), which results in significant alterations in the biosynthesis of key monoamines (e.g., serotonin, dopamine) known to play a major role in mood regulation and cognitive function (Dantzer et al., 2008; Capuron et al., 2011b). As part of the immune response to infection, cytokine-induced activation of IDO, which is the first and rate-limiting enzyme degrading tryptophan along the kynurenine pathway, is usually beneficial to the host (Mellor and Munn, 2008). However, sustained brain IDO activation can also be deleterious by negatively impacting monoaminergic neurotransmission, but also neuronal survival. Interestingly, development of neuropsychiatric symptoms in medically ill patients chronically treated with IFN-α (Raison et al., 2010), elderly subjects (Capuron et al., 2011b), and patients suffering from Alzheimer's disease (Gulaj et al., 2010) or strokes (Gold et al., 2011) is associated with increased circulating levels of kynurenine. Severely obese individuals with high prevalence of neuropsychiatric comorbidity also display activation of IDO (Brandacher et al., 2006, 2007), together with larger hippocampal atrophy than non-obese subjects (Fotuhi et al., 2012). Increased brain kynurenine levels resulting from IDO activation can be further metabolized to produce neuroactive glutamatergic compounds, including 3-hydroxykynurenine (3HKyn) and quinolinic acid (QA), which play a key role in neuronal death and neurodegenerative diseases by stimulating NMDA receptors and promoting oxidative stress (Figure 1) (Stone et al., 2012; Campbell et al., 2014). On the other hand, kynurenine can also be metabolized in kynurenic acid (KA) that rather displays neuroprotective properties. However, these apparently antagonistic pathways are compartmentalized in the brain, with microglia preferentially producing QA, whereas astrocytes produce KA. Sustained immune activation therefore tips the scale in favor of neurotoxicity. In agreement with this finding, increased brain, or cerebrospinal fluid (CSF) concentrations of kynurenine and its neurotoxic metabolites have been reported in several neurodegenerative and psychiatric disorders (Schwarcz et al., 2001; Myint et al., 2007; Stone et al., 2012; Campbell et al., 2014). Moreover, they have been related with the stretch of brain damages, and with mood and cognitive impairments (Stone et al., 2012), suggesting that IDO activation may lead to both functional and structural alterations in the brain. Consistent with this assumption, activation of the kynurenine pathway has been recently shown to affect human hippocampal neurogenesis (Zunszain et al., 2012; Savitz et al., 2015a,b).

In line with clinical findings, experimental studies showed that development of depressive-like and anxiety-like behaviors induced by acute or chronic immune challenges in mice (Frenois et al., 2007; Godbout et al., 2008; Moreau et al., 2008; O'Connor et al., 2009a,b,c; Salazar et al., 2012; Corona et al., 2013; Lawson et al., 2013) is associated with increased peripheral and brain IDO activity (Lestage et al., 2002; Moreau et al., 2005; André et al., 2008). Similarly, brain IDO activation was also associated with cognitive and emotional alterations following immune activation (Lawson et al., 2011; Barichello et al., 2013; Gibney et al., 2013; Xie et al., 2014). More importantly, pharmacological, or genetic inhibition of IDO activity prevents induction of depressive-like behaviors, anxiety-like behaviors and/or cognitive impairments by systemic immune challenges, without impacting sickness behavior (Henry et al., 2009; O'Connor et al., 2009a,b,c; Salazar et al., 2012; Barichello et al., 2013; Xie et al., 2014). In addition, aged mice (Godbout et al., 2008; Kelley et al., 2013), mice exhibiting constitutive microglial over-activation (Corona et al., 2010) and obese mice (André et al., 2014) display sustained cytokine production after an immune challenge, together with protracted brain IDO expression and depressive-like behavior (Godbout et al., 2008; Wynne et al., 2010; Corona et al., 2013). Further studies report that centrally induced inflammation, which only activates brain kynurenine pathway, is sufficient to elicit depressive-like behaviors (Fu et al., 2010; Park et al., 2011; Dobos et al., 2012; Lawson et al., 2013). In addition, other studies shed light on the hippocampus as important brain area for activation of cytokines and IDO (Frenois et al., 2007; André et al., 2008; Henry et al., 2009; Wang et al., 2009; Corona et al., 2010; Fu et al., 2010), although they are broadly stimulated within the brain in response to immune challenges (Castanon et al., 2004; André et al., 2008). Interestingly, emotional alterations linked to hippocampus IDO activation by an immune challenge are associated with reduced hippocampal expression of the brain-derived neurotrophic factor (BDNF) (Gibney et al., 2013). This neurotrophin contributes to mood regulation and memory function, including in conditions of immune activation (Barrientos et al., 2004), by supporting synaptic plasticity and neuronal excitability (Yamada and Nabeshima, 2003; Martinowich et al., 2007). Interestingly, cognitive impairment and emotional alterations reported in both obese DIO mice and *db/db* mice are also associated with reduced BDNF levels in the cortex (Pistell et al., 2010) and the hippocampus (Dinel et al., 2011). Reciprocally, interventions normalizing hippocampal levels of BDNF in these mice prevent hippocampus-mediated cognitive impairments (Moy and McNay, 2012; Kariharan et al., 2015). These results point to a link between increased neuroinflammation, impaired neurogenesis/synaptic plasticity, and behavioral alterations in obesity. Of note, brain IDO activation results in obese mice in a huge increase of brain kynurenine concentrations compared to lean mice, but similar impairment of brain tryptophan levels (André et al., 2014). These results support those showing that administration of kynurenine dose-dependently induces depressive-like behaviors, anxiety-like behaviors and cognitive impairment in normal-weight mice (Chess et al., 2009; O'Connor et al., 2009c; Alexander et al., 2012; Salazar et al., 2012; Agudelo et al., 2014). Altogether, although these results do not exclude the possible role of impaired serotonin synthesis in inflammation-induced neuropsychiatric symptoms, they clearly support a key role for the neuroactive kynurenine metabolites resulting from IDO activation.

**Figure 2:**
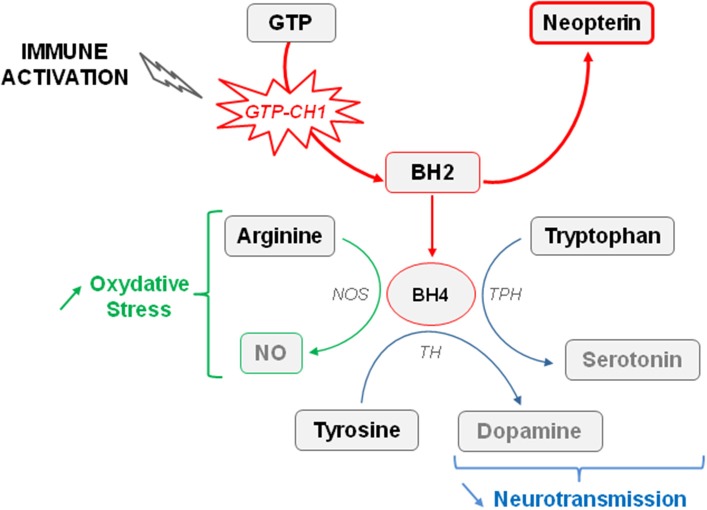
**Effect of immune activation on the availability of tetrahydrobiopterin (BH4)**. The guanosine-triphosphate-cyclohydrolase-1 (GTP-CH1) is the rate limiting enzyme of guanosine-triphosphate (GTP) conversion in dihydrobiopterin (BH2). Increased GTP-CH1 activity occurring in conditions of immune activation ultimately leads to the production of neopterin at the expense of tetrahydrobiopterin (BH4) formation. BH4 plays a fundamental role in neurotransmitter synthesis, in particular serotonin and dopamine, by acting as a cofactor of the tryptophan hydroxylase (TPH) and tyrosine hydroxylase (TH). Cytokine-induced GTP-CH1 activation reduces therefore monoaminergic neurotransmission. BH4 is also a cofactor of the nitric oxide synthase (NOS). Reduced BH4 availability can indirectly contributes to increase the production of free radicals promoting oxidative stress.

Concurrently with activation of IDO, chronic inflammation also activates GTP-CH1, which is the rate limiting enzyme of GTP conversion ultimately leading to the production of neopterin by activated immune cells at the expense of tetrahydrobiopterin (BH4) formation (Figure 2) (Murr et al., 2002; Oxenkrug, 2010; Capuron et al., 2011b). By acting as a cofactor of aromatic amino acid hydroxylase, BH4 plays a fundamental role in neurotransmitter synthesis, including serotonin (Capuron et al., 2011b) but mainly dopamine (Neurauter et al., 2008). Cytokine-induced GTP-CH1 activation, classically assessed by measuring increased production of neopterin, is indeed able to impair the dopaminergic neurotransmission (Felger and Miller, 2012) which is known to be involved in mood disorders and cognitive dysfunctions, including in conditions of chronic immune stimulation (Brydon et al., 2008; Capuron et al., 2012). Consistent with these findings, reduced BH4 levels have been reported in patients with psychiatric disorders (Hashimoto et al., 1994; Hoekstra et al., 2001). Similarly, increased blood neopterin concentrations correlate with a greater incidence of depressive episodes in patients with major depression (Celik et al., 2010). Interestingly, chronic low-grade inflammation that is classically reported in elderly people is associated with activation of both IDO and GTP-CH1 (Oxenkrug, 2010; Capuron et al., 2011b). More importantly, these enzymatic activations have been shown to participate in the pathophysiology of neuropsychiatric symptoms, whose prevalence is often high in aged population (Oxenkrug, 2010; Capuron et al., 2011b). Of note, in addition to impair monoaminergic neurotransmission, both enzymes contributes to increase oxidative stress (Felger and Miller, 2012; Stone et al., 2012; Campbell et al., 2014). Increased neopterin levels have been reported in obese rats (Finkelstein et al., 1982) and humans (Ledochowski et al., 1999; Oxenkrug et al., 2011), suggesting that activation of the GTP-CH1 enzyme by cytokines, and the consequent alterations of dopamine neurotransmission, may contribute to the development of neuropsychiatric symptoms reported in obesity. Although this assumption still needs to be confirmed, it is worth mentioning that alterations of dopamine function, together with alterations in basal ganglia/reward circuitry have been reported in obese patients (de Weijer et al., 2011; Volkow et al., 2011). Moreover, depressive-like behavior is associated in DIO mice with alterations in striatal circuitry, supporting a role for dopamine-related disruptions in obesity-associated depressive symptoms (Sharma and Fulton, 2013).

## Factors modulating the impact of neuroinflammation on cognitive and emotional alterations associated with obesity

### HPA axis

Brain effects of cytokines on mood regulation and cognitive function are likely modulated by the tight interactions existing between the inflammatory and neuroendocrine systems, in particular the HPA axis (Raison and Miller, 2003; Capuron and Miller, 2011) which is highly activated in obesity (Dinel et al., 2011, 2014). Immune alterations are indeed notorious for causing significant changes in HPA axis activity and *vice versa*. Consequently, alterations of HPA axis functions and inflammatory activation are often associated in many pathological conditions, including cancers treated by immunotherapy (Capuron et al., 2003), mood disorders (Zunszain et al., 2011) or obesity (Dinel et al., 2011, 2014; André et al., 2014). Interestingly, glucocorticoids have been recently shown to sensitize microglia in an animal model of obesity (Dey et al., 2014). Reciprocally, DIO mice display exacerbated HPA axis activation in response to an immune challenge, together with increased neuroinflammation and depressive-like behavior (André et al., 2014). This result supports the notion that inflammatory factors and glucocorticoids may act together in the context of obesity to promote the development of depressive symptoms (Hryhorczuk et al., 2013) and cognitive functions, in particular those involving the hippocampus (Stranahan et al., 2008; Dey et al., 2014). Interestingly, both cytokines and glucocorticoids have been shown for example to impair hippocampal neurogenesis and neuronal function in animal models of obesity (Stranahan et al., 2008; Dinel et al., 2011; Erion et al., 2014; Wosiski-Kuhn et al., 2014). Moreover, reversing the impairment of hippocampal neurogenesis by targeting cytokines or glucocorticoids improves spatial memory deficits displayed by obese mice (Stranahan et al., 2008; Erion et al., 2014).

### Leptin

One of the stronger features associated with obesity is the significant increase of leptin production (Lehr et al., 2012). This adipokine has been extensively studied for decades for its key role in the control of energy homeostasis and feeding behavior through activation of specific leptin receptors in the hypothalamus (Rosenbaum and Leibel, 2014). However, due to the broad brain distribution of leptin receptors, which are also located throughout the cortex and the hippocampus, leptin has been shown to modulate memory processes and mood disorders (Guo et al., 2013; Farr et al., 2015). Leptin is also known to locally facilitate synaptic plasticity and neurogenesis (Shanley et al., 2001). Deficits in spatial memory reported in DIO mice occur concomitantly with desensitization of leptin signaling pathway in the hippocampus (Valladolid-Acebes et al., 2013). Moreover, selective deletion of leptin receptors in adult hippocampus induces depressive-like behavior in mice (Guo et al., 2013) and confers resistance to behavioral antidepressant effect of fluoxetine (Guo and Lu, 2014). In addition, obese mice which are characterized by impaired leptin signaling pathway (e.g., *db/db* or *ob/ob* mice) display deficits of hippocampal-dependent memory and increased anxiety-like behavior (Stranahan et al., 2008; Dinel et al., 2011), together with increased basal hippocampal inflammation (Dinel et al., 2011; Erion et al., 2014) and altered neuroinflammatory response to an immune challenge (Rummel et al., 2010; Lawrence et al., 2012; Dinel et al., 2014). These findings suggest that both leptin and cytokines may contribute together to the development of behavioral alterations associated with obesity. Interestingly, this assumption is supported by mounting evidence showing that leptin acts as an important neuroendocrine modulator of the immune system by modulating the activation of both peripheral immune cells and brain microglia (Lafrance et al., 2010; Aguilar-Valles et al., 2015).

### Insulin

This hormone, whose circulating levels and signaling pathway are altered in obesity, is able to interact with inflammatory processes and to act within the brain to modulate mood and cognition (Ghasemi et al., 2013). Impaired insulin signaling pathway may therefore, as with leptin, contribute to the development of neuropsychiatric symptoms in the context of obesity by interacting with brain cytokines. More studies are necessary to test this hypothesis. However, it is worth noting that several studies including ours suggest that insulin *per se* is not essential or sufficient to explain behavioral alterations occurring in obesity. For example, the increased risk of cognitive dysfunction displayed by patients with metabolic syndrome is independent from the presence of diabetes (Muller et al., 2010). Consistent with these clinical findings, we recently reported increased emotional behaviors and cognitive impairment in DIO animals in the absence of any significant hyperinsulinemia (André et al., 2014; Boitard et al., 2014). Similarly, normalization of peripheral hyperglycemia in *db/db* mice does not improve spatial cognitive impairments or anxiety-like behaviors (Stranahan et al., 2008, 2009). Besides, this normalization does not alter brain concentrations of glucose and insulin that are similar in both *db/db* and *db/+* mice (Stranahan et al., 2008).

### Gut microbiota

Converging evidence shows that obesity-related microbiota dysregulations, which play a critical role in induction of peripheral and brain inflammation (Bruce-Keller et al., 2015), impact mood and cognitive functions (Cryan and Dinan, 2012), although the nature of the biological pathways (neuronal, hormonal and/or immune) underlying this effect is still elusive (Cryan and Dinan, 2012). Interestingly, it has been recently reported that transplantation of gut microbiota from DIO mice to lean mice is sufficient to induce both brain microglial activation and neurobehavioral changes in the absence of obesity (Bruce-Keller et al., 2015). This elegant study supports the notion that obesity-related gut microbiota alterations may modulate gut-to-brain communication pathways, leading to the development of neuropsychiatric comorbidities associated with neuroinflammation. Akin with this assumption, the use of compounds which beneficially alter the microbiota (e.g., prebiotics or probiotics) appears as a promising way to improve neuropsychiatric comorbidities in obese patients (Cryan and Dinan, 2012). More generally, nutritional interventions based on factors with immunomodulatory properties and known impact on behavior and mood, in particular omega-3 polyunsaturated fatty acids and antioxidants (see Gomez-Pinilla and Nguyen, 2012; Bazinet and Layé, 2014 for review), are tractable strategies for developing novel therapeutics for obesity-related neuropsychiatric disorders. Lastly, based on the key role of the kynurenine pathway in altering mood and cognition (Dantzer et al., 2008), the opportunity of directly targeting this pathway, as promisingly tested in the context of cancer (Platten et al., 2015), should likely represent another interesting therapeutic approach.

## Conclusion

**Figure 3:**
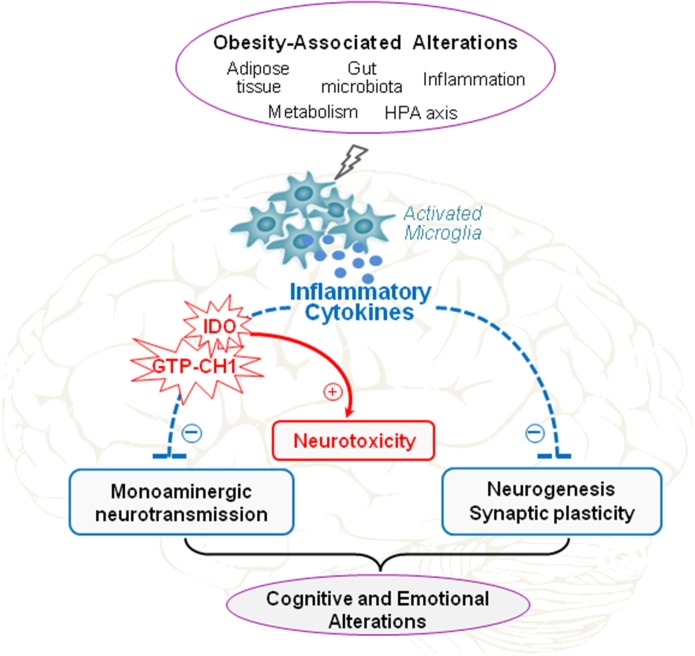
**Proposed role of neuroinflammation in obesity-associated cognitive and emotional alterations**. Obesity is characterized by metabolic alterations (hyperinsulinemia, hyperleptinemia, increased activation of the hypothalamo-pituitary-adrenal (HPA) axis…) and peripheral low-grade inflammation, which originates from alterations in adipose tissue and gut functions. These obesity-associated alterations are well-known to promote brain inflammatory processes that represent key players in the development of behavioral alterations associated with obesity. By sustaining neuroinflammation, as manifested by chronic activation of microglia, brain production of inflammatory cytokines, and local activation of indoleamine 2,3-dioxygenase (IDO) and guanosine-triphosphate-cyclohydrolase-1 (GTP-CH1), obesity may impair monoaminergic neurotransmission, neurogenesis, and synaptic plasticity, and concomitantly promote neurotoxicity. Such alterations of brain function induced by neuroinflammation likely represent major pathophysiological pathways to cognitive and emotional alterations in obesity.

Altogether, data presented in this review clearly show that brain inflammatory processes represent key players in the development of neuropsychiatric comorbidities in obesity. These likely rely on interactions involving multiple systems, including inflammatory processes but also neuroendocrine systems, gut microbiota and environmental influences. Indeed, neuroinflammation appears to be the cornerstone of the different factors contributing to induce neuropsychiatric symptoms in obesity. Several issues are however still unclear, in particular the identification of the specific brain pathways and/or mechanisms targeted by neuroinflammation and underlying obesity-related mood and cognitive alterations. Recent experimental results reported in this review suggest that cytokine-induced unbalanced relationship between neurogenesis and neuronal death combined with alterations in monoamine metabolism and function likely represent a major pathophysiological pathway to neuropsychiatric comorbidities in obesity (Figure 3). Given the alarming and continuous rise in obesity in modern societies and its role as a risk factor for many other diseases including neuropsychiatric disorders, therapeutic strategies to reduce obesity-related inflammation may be very promising approach to improve the quality of life and health outcomes of overweight and obese individuals.

### Conflict of interest statement

The authors declare that the research was conducted in the absence of any commercial or financial relationships that could be construed as a potential conflict of interest.

## References

[B1] Agudelo L. Z. Femenia T. Orhan F. Porsmyr-Palmertz M. Goiny M. Martinez-Redondo V.. (2014). Skeletal muscle PGC-1alpha1 modulates kynurenine metabolism and mediates resilience to stress-induced depression. Cell 159, 33–45. 10.1016/j.cell.2014.07.051 25259918

[B2] Aguilar-Valles A. Inoue W. Rummel C. Luheshi G. N. (2015). Obesity, adipokines and neuroinflammation. Neuropharmacology 96, 124–134. 10.1016/j.neuropharm.2014.12.023 25582291

[B3] Aguilar-Valles A. Kim J. Jung S. Woodside B. Luheshi G. N. (2014). Role of brain transmigrating neutrophils in depression-like behavior during systemic infection. Mol. Psychiatry 19, 599–606. 10.1038/mp.2013.137 24126927

[B4] Alexander K. S. Wu H. Q. Schwarcz R. Bruno J. P. (2012). Acute elevations of brain kynurenic acid impair cognitive flexibility: normalization by the alpha7 positive modulator galantamine. Psychopharmacology (Berl.) 220, 627–637. 10.1007/s00213-011-2539-2 22038535 PMC3666324

[B5] Alosco M. L. Spitznagel M. B. Strain G. Devlin M. Cohen R. Paul R.. (2014). Improved memory function two years after bariatric surgery. Obesity (Silver Spring) 22, 32–38. 10.1002/oby.20494 23625587 PMC4054602

[B6] Amar S. Zhou Q. Shaik-Dasthagirisaheb Y. Leeman S. (2007). Diet-induced obesity in mice causes changes in immune responses and bone loss manifested by bacterial challenge. Proc. Natl. Acad. Sci. U.S.A. 104, 20466–20471. 10.1073/pnas.0710335105 18077329 PMC2154454

[B7] Andersen J. R. Aasprang A. Bergsholm P. Sletteskog N. Vage V. Natvig G. K. (2010). Anxiety and depression in association with morbid obesity: changes with improved physical health after duodenal switch. Health Qual. Life Outcomes 8:52. 10.1186/1477-7525-8-52 20492663 PMC2881107

[B8] André C. Dinel A. L. Ferreira G. Laye S. Castanon N. (2014). Diet-induced obesity progressively alters cognition, anxiety-like behavior and lipopolysaccharide-induced depressive-like behavior: focus on brain indoleamine 2,3-dioxygenase activation. Brain Behav. Immun. 41, 10–21. 10.1016/j.bbi.2014.03.012 24681251

[B9] André C. O'Connor J. C. Kelley K. W. Lestage J. Dantzer R. Castanon N. (2008). Spatio-temporal differences in the profile of murine brain expression of proinflammatory cytokines and indoleamine 2,3-dioxygenase in response to peripheral lipopolysaccharide administration. J. Neuroimmunol. 200, 90–99. 10.1016/j.jneuroim.2008.06.011 18653240 PMC2571040

[B10] Barichello T. Generoso J. S. Simoes L. R. Elias S. G. Tashiro M. H. Dominguini D.. (2013). Inhibition of indoleamine 2,3-dioxygenase prevented cognitive impairment in adult Wistar rats subjected to pneumococcal meningitis. Transl. Res. 162, 390–397. 10.1016/j.trsl.2013.08.001 23994082

[B11] Barrientos R. M. Sprunger D. B. Campeau S. Watkins L. R. Rudy J. W. Maier S. F. (2004). BDNF mRNA expression in rat hippocampus following contextual learning is blocked by intrahippocampal IL-1beta administration. J. Neuroimmunol. 155, 119–126. 10.1016/j.jneuroim.2004.06.009 15342202

[B12] Bazinet R. P. Layé S. (2014). Polyunsaturated fatty acids and their metabolites in brain function and disease. Nat. Rev. Neurosci. 15, 771–785. 10.1038/nrn3820 25387473

[B13] Belza A. Toubro S. Stender S. Astrup A. (2009). Effect of diet-induced energy deficit and body fat reduction on high-sensitive CRP and other inflammatory markers in obese subjects. Int. J. Obes. (Lond.) 33, 456–464. 10.1038/ijo.2009.27 19238154

[B14] Biessels G. J. Gispen W. H. (2005). The impact of diabetes on cognition: what can be learned from rodent models? Neurobiol. Aging 26(Suppl. 1), 36–41. 10.1016/j.neurobiolaging.2005.08.015 16223548

[B15] Bilbo S. D. Tsang V. (2010). Enduring consequences of maternal obesity for brain inflammation and behavior of offspring. FASEB J. 24, 2104–2115. 10.1096/fj.09-144014 20124437

[B16] Bluher M. Mantzoros C. S. (2015). From leptin to other adipokines in health and disease: facts and expectations at the beginning of the 21st century. Metab. Clin. Exp. 64, 131–145. 10.1016/j.metabol.2014.10.016 25497344

[B17] Boitard C. Cavaroc A. Sauvant J. Aubert A. Castanon N. Laye S.. (2014). Impairment of hippocampal-dependent memory induced by juvenile high-fat diet intake is associated with enhanced hippocampal inflammation in rats. Brain Behav. Immun. 40, 9–17. 10.1016/j.bbi.2014.03.005 24662056

[B18] Boitard C. Etchamendy N. Sauvant J. Aubert A. Tronel S. Marighetto A.. (2012). Juvenile, but not adult exposure to high-fat diet impairs relational memory and hippocampal neurogenesis in mice. Hippocampus 22, 2095–2100. 10.1002/hipo.22032 22593080

[B19] Brandacher G. Hoeller E. Fuchs D. Weiss H. G. (2007). Chronic immune activation underlies morbid obesity: is IDO a key player? Curr. Drug Metab. 8, 289–295. 10.2174/138920007780362590 17430117

[B20] Brandacher G. Winkler C. Aigner F. Schwelberger H. Schroecksnadel K. Margreiter R.. (2006). Bariatric surgery cannot prevent tryptophan depletion due to chronic immune activation in morbidly obese patients. Obes. Surg. 16, 541–548. 10.1381/096089206776945066 16687019

[B21] Brinkworth G. D. Buckley J. D. Noakes M. Clifton P. M. Wilson C. J. (2009). Long-term effects of a very low-carbohydrate diet and a low-fat diet on mood and cognitive function. Arch. Intern. Med. 169, 1873–1880. 10.1001/archinternmed.2009.329 19901139

[B22] Bruce-Keller A. J. Salbaum J. M. Luo M. Blanchard E. T. Taylor C. M. Welsh D. A.. (2015). Obese-type gut microbiota induce neurobehavioral changes in the absence of obesity. Biol. Psychiatry 77, 607–615. 10.1016/j.biopsych.2014.07.012 25173628 PMC4297748

[B23] Brun P. Castagliuolo I. Di Leo V. Buda A. Pinzani M. Palu G.. (2007). Increased intestinal permeability in obese mice: new evidence in the pathogenesis of nonalcoholic steatohepatitis. Am. J. Physiol. Gastrointest. Liver Physiol. 292, G518–G525. 10.1152/ajpgi.00024.2006 17023554

[B24] Brydon L. Harrison N. A. Walker C. Steptoe A. Critchley H. D. (2008). Peripheral inflammation is associated with altered substantia nigra activity and psychomotor slowing in humans. Biol. Psychiatry 63, 1022–1029. 10.1016/j.biopsych.2007.12.007 18242584 PMC2885493

[B25] Buckman L. B. Hasty A. H. Flaherty D. K. Buckman C. T. Thompson M. M. Matlock B. K.. (2014). Obesity induced by a high-fat diet is associated with increased immune cell entry into the central nervous system. Brain Behav. Immun. 35, 33–42. 10.1016/j.bbi.2013.06.007 23831150 PMC3858467

[B26] Cai D. Liu T. (2012). Inflammatory cause of metabolic syndrome via brain stress and NF-kappaB. Aging (Albany NY) 4, 98–115. 22328600 10.18632/aging.100431 PMC3314172

[B27] Campbell B. M. Charych E. Lee A. W. Moller T. (2014). Kynurenines in CNS disease: regulation by inflammatory cytokines. Front. Neurosci. 8:12. 10.3389/fnins.2014.00012 24567701 PMC3915289

[B28] Cancello R. Clement K. (2006). Is obesity an inflammatory illness? Role of low-grade inflammation and macrophage infiltration in human white adipose tissue. BJOG 113, 1141–1147. 10.1111/j.1471-0528.2006.01004.x 16903845

[B29] Cani P. D. Amar J. Iglesias M. A. Poggi M. Knauf C. Bastelica D.. (2007). Metabolic endotoxemia initiates obesity and insulin resistance. Diabetes 56, 1761–1772. 10.2337/db06-1491 17456850

[B30] Cani P. D. Bibiloni R. Knauf C. Waget A. Neyrinck A. M. Delzenne N. M.. (2008). Changes in gut microbiota control metabolic endotoxemia-induced inflammation in high-fat diet-induced obesity and diabetes in mice. Diabetes 57, 1470–1481. 10.2337/db07-1403 18305141

[B31] Cani P. D. Osto M. Geurts L. Everard A. (2012). Involvement of gut microbiota in the development of low-grade inflammation and type 2 diabetes associated with obesity. Gut Microbes 3, 279–288. 10.4161/gmic.19625 22572877 PMC3463487

[B32] Cani P. D. Possemiers S. van de Wiele T. Guiot Y. Everard A. Rottier O.. (2009). Changes in gut microbiota control inflammation in obese mice through a mechanism involving GLP-2-driven improvement of gut permeability. Gut 58, 1091–1103. 10.1136/gut.2008.165886 19240062 PMC2702831

[B33] Capuron L. Miller A. H. (2011). Immune system to brain signaling: neuropsychopharmacological implications. Pharmacol. Ther. 130, 226–238. 10.1016/j.pharmthera.2011.01.014 21334376 PMC3072299

[B34] Capuron L. Pagnoni G. Drake D. F. Woolwine B. J. Spivey J. R. Crowe R. J.. (2012). Dopaminergic mechanisms of reduced basal ganglia responses to hedonic reward during interferon alfa administration. Arch. Gen. Psychiatry 69, 1044–1053. 10.1001/archgenpsychiatry.2011.2094 23026954 PMC3640298

[B35] Capuron L. Poitou C. Machaux-Tholliez D. Frochot V. Bouillot J. L. Basdevant A.. (2011a). Relationship between adiposity, emotional status and eating behaviour in obese women: role of inflammation. Psychol. Med. 41, 1517–1528. 10.1017/S0033291710001984 20961476

[B36] Capuron L. Raison C. L. Musselman D. L. Lawson D. H. Nemeroff C. B. Miller A. H. (2003). Association of exaggerated HPA axis response to the initial injection of interferon-alpha with development of depression during interferon-alpha therapy. Am. J. Psychiatry 160, 1342–1345. 10.1176/appi.ajp.160.7.1342 12832253

[B37] Capuron L. Schroecksnadel S. Feart C. Aubert A. Higueret D. Barberger-Gateau P.. (2011b). Chronic low-grade inflammation in elderly persons is associated with altered tryptophan and tyrosine metabolism: role in neuropsychiatric symptoms. Biol. Psychiatry 70, 175–182. 10.1016/j.biopsych.2010.12.006 21277567

[B38] Capuron L. Su S. Miller A. H. Bremner J. D. Goldberg J. Vogt G. J.. (2008). Depressive symptoms and metabolic syndrome: is inflammation the underlying link? Biol. Psychiatry 64, 896–900. 10.1016/j.biopsych.2008.05.019 18597739 PMC2621309

[B39] Castanon N. Lasselin J. Capuron L. (2014). Neuropsychiatric comorbidity in obesity: role of inflammatory processes. Front. Endocrinol. (Lausanne) 5:74. 10.3389/fendo.2014.00074 24860551 PMC4030152

[B40] Castanon N. Leonard B. E. Neveu P. J. Yirmiya R. (2002). Effects of antidepressants on cytokine production and actions. Brain Behav. Immun. 16, 569–574. 10.1016/S0889-1591(02)00008-9 12401470

[B41] Castanon N. Medina C. Mormede C. Dantzer R. (2004). Chronic administration of tianeptine balances lipopolysaccharide-induced expression of cytokines in the spleen and hypothalamus of rats. Psychoneuroendocrinology 29, 778–790. 10.1016/S0306-4530(03)00142-2 15110927

[B42] Celik C. Erdem M. Cayci T. Ozdemir B. Ozgur Akgul E. Kurt Y. G.. (2010). The association between serum levels of neopterin and number of depressive episodes of major depression. Prog. Neuropsychopharmacol. Biol. Psychiatry 34, 372–375. 10.1016/j.pnpbp.2010.01.002 20074610

[B43] Chess A. C. Landers A. M. Bucci D. J. (2009). L-kynurenine treatment alters contextual fear conditioning and context discrimination but not cue-specific fear conditioning. Behav. Brain Res. 201, 325–331. 10.1016/j.bbr.2009.03.013 19428652

[B44] Collin M. Hakansson-Ovesjo M. L. Misane I. Ogren S. O. Meister B. (2000). Decreased 5-HT transporter mRNA in neurons of the dorsal raphe nucleus and behavioral depression in the obese leptin-deficient ob/ob mouse. Brain Res. Mol. Brain Res. 81, 51–61. 10.1016/S0169-328X(00)00167-4 11000478

[B45] Corona A. W. Huang Y. O'Connor J. C. Dantzer R. Kelley K. W. Popovich P. G.. (2010). Fractalkine receptor (CX3CR1) deficiency sensitizes mice to the behavioral changes induced by lipopolysaccharide. J. Neuroinflammation 7:93. 10.1186/1742-2094-7-93 21167054 PMC3018416

[B46] Corona A. W. Norden D. M. Skendelas J. P. Huang Y. O'Connor J. C. Lawson M.. (2013). Indoleamine 2,3-dioxygenase inhibition attenuates lipopolysaccharide induced persistent microglial activation and depressive-like complications in fractalkine receptor (CX(3)CR1)-deficient mice. Brain Behav. Immun. 31, 134–142. 10.1016/j.bbi.2012.08.008 22926082 PMC3554840

[B47] Cournot M. Marquie J. C. Ansiau D. Martinaud C. Fonds H. Ferrieres J.. (2006). Relation between body mass index and cognitive function in healthy middle-aged men and women. Neurology 67, 1208–1214. 10.1212/01.wnl.0000238082.13860.50 17030754

[B48] Creely S. J. McTernan P. G. Kusminski C. M. Fisher F. M. Da Silva N. F. Khanolkar M.. (2007). Lipopolysaccharide activates an innate immune system response in human adipose tissue in obesity and type 2 diabetes. Am. J. Physiol. Endocrinol. Metab. 292, E740–E747. 10.1152/ajpendo.00302.2006 17090751

[B49] Cryan J. F. Dinan T. G. (2012). Mind-altering microorganisms: the impact of the gut microbiota on brain and behaviour. Nat. Rev. Neurosci. 13, 701–712. 10.1038/nrn3346 22968153

[B50] Dahl A. K. Hassing L. B. Fransson E. I. Gatz M. Reynolds C. A. Pedersen N. L. (2013). Body mass index across midlife and cognitive change in late life. Int. J. Obes. (Lond.) 37, 296–302. 10.1038/ijo.2012.37 22450854 PMC3387354

[B51] Dantzer R. O'Connor J. C. Freund G. G. Johnson R. W. Kelley K. W. (2008). From inflammation to sickness and depression: when the immune system subjugates the brain. Nat. Rev. Neurosci. 9, 46–56. 10.1038/nrn2297 18073775 PMC2919277

[B52] De Souza C. T. Araujo E. P. Bordin S. Ashimine R. Zollner R. L. Boschero A. C.. (2005). Consumption of a fat-rich diet activates a proinflammatory response and induces insulin resistance in the hypothalamus. Endocrinology 146, 4192–4199. 10.1210/en.2004-1520 16002529

[B53] de Weijer B. A. van de Giessen E. van Amelsvoort T. A. Boot E. Braak B. Janssen I. M.. (2011). Lower striatal dopamine D2/3 receptor availability in obese compared with non-obese subjects. EJNMMI Res. 1:37. 10.1186/2191-219X-1-37 22214469 PMC3265412

[B54] Dey A. Hao S. Erion J. R. Wosiski-Kuhn M. Stranahan A. M. (2014). Glucocorticoid sensitization of microglia in a genetic mouse model of obesity and diabetes. J. Neuroimmunol. 269, 20–27. 10.1016/j.jneuroim.2014.01.013 24534266 PMC3989932

[B55] Dinel A. L. Andre C. Aubert A. Ferreira G. Laye S. Castanon N. (2011). Cognitive and emotional alterations are related to hippocampal inflammation in a mouse model of metabolic syndrome. PLoS ONE 6:e24325. 10.1371/journal.pone.0024325 21949705 PMC3174932

[B56] Dinel A. L. Andre C. Aubert A. Ferreira G. Laye S. Castanon N. (2014). Lipopolysaccharide-induced brain activation of the indoleamine 2,3-dioxygenase and depressive-like behavior are impaired in a mouse model of metabolic syndrome. Psychoneuroendocrinology 40, 48–59. 10.1016/j.psyneuen.2013.10.014 24485475

[B57] Dobos N. de Vries E. F. Kema I. P. Patas K. Prins M. Nijholt I. M.. (2012). The role of indoleamine 2,3-dioxygenase in a mouse model of neuroinflammation-induced depression. J. Alzheimers Dis. 28, 905–915. 10.3233/JAD-2011-111097 22112548

[B58] Emery C. F. Fondow M. D. Schneider C. M. Christofi F. L. Hunt C. Busby A. K.. (2007). Gastric bypass surgery is associated with reduced inflammation and less depression: a preliminary investigation. Obes. Surg. 17, 759–763. 10.1007/s11695-007-9140-0 17879575

[B59] Erion J. R. Wosiski-Kuhn M. Dey A. Hao S. Davis C. L. Pollock N. K.. (2014). Obesity elicits interleukin 1-mediated deficits in hippocampal synaptic plasticity. J. Neurosci. 34, 2618–2631. 10.1523/JNEUROSCI.4200-13.2014 24523551 PMC3921429

[B60] Evans D. L. Charney D. S. Lewis L. Golden R. N. Gorman J. M. Krishnan K. R.. (2005). Mood disorders in the medically ill: scientific review and recommendations. Biol. Psychiatry 58, 175–189. 10.1016/j.biopsych.2005.05.001 16084838

[B61] Farooqui A. A. Farooqui T. Panza F. Frisardi V. (2012). Metabolic syndrome as a risk factor for neurological disorders. Cell. Mol. Life Sci. 69, 741–762. 10.1007/s00018-011-0840-1 21997383 PMC11115054

[B62] Farr O. M. Tsoukas M. A. Mantzoros C. S. (2015). Leptin and the brain: influences on brain development, cognitive functioning and psychiatric disorders. Metab. Clin. Exp. 64, 114–130. 10.1016/j.metabol.2014.07.004 25092133

[B63] Felger J. C. Miller A. H. (2012). Cytokine effects on the basal ganglia and dopamine function: the subcortical source of inflammatory malaise. Front. Neuroendocrinol. 33, 315–327. 10.1016/j.yfrne.2012.09.003 23000204 PMC3484236

[B64] Fiedorowicz J. G. Palagummi N. M. Forman-Hoffman V. L. Miller D. D. Haynes W. G. (2008). Elevated prevalence of obesity, metabolic syndrome, and cardiovascular risk factors in bipolar disorder. Ann. Clin. Psychiatry 20, 131–137. 10.1080/10401230802177722 18633739 PMC2776768

[B65] Finelli C. Padula M. C. Martelli G. Tarantino G. (2014). Could the improvement of obesity-related co-morbidities depend on modified gut hormones secretion? World J. Gastroenterol. 20, 16649–16664. 10.3748/wjg.v20.i44.16649 25469034 PMC4248209

[B66] Finkelstein J. A. Chance W. T. Fischer J. E. (1982). Brain serotonergic activity and plasma amino acid levels in genetically obese Zucker rats. Pharmacol. Biochem. Behav. 17, 939–944. 10.1016/0091-3057(82)90476-2 6184736

[B67] Fotuhi M. Do D. Jack C. (2012). Modifiable factors that alter the size of the hippocampus with ageing. Nat. Rev. Neurol. 8, 189–202. 10.1038/nrneurol.2012.27 22410582

[B68] Francis H. Stevenson R. (2013). The longer-term impacts of Western diet on human cognition and the brain. Appetite 63, 119–128. 10.1016/j.appet.2012.12.018 23291218

[B69] Frenois F. Moreau M. O'Connor J. Lawson M. Micon C. Lestage J.. (2007). Lipopolysaccharide induces delayed FosB/DeltaFosB immunostaining within the mouse extended amygdala, hippocampus and hypothalamus, that parallel the expression of depressive-like behavior. Psychoneuroendocrinology 32, 516–531. 10.1016/j.psyneuen.2007.03.005 17482371 PMC1978247

[B70] Frisardi V. Solfrizzi V. Seripa D. Capurso C. Santamato A. Sancarlo D.. (2010). Metabolic-cognitive syndrome: a cross-talk between metabolic syndrome and Alzheimer's disease. Ageing Res. Rev. 9, 399–417. 10.1016/j.arr.2010.04.007 20444434

[B71] Fu X. Zunich S. M. O'Connor J. C. Kavelaars A. Dantzer R. Kelley K. W. (2010). Central administration of lipopolysaccharide induces depressive-like behavior *in vivo* and activates brain indoleamine 2,3 dioxygenase in murine organotypic hippocampal slice cultures. J. Neuroinflammation 7:43. 10.1186/1742-2094-7-43 20678226 PMC2921406

[B72] Gao Y. Ottaway N. Schriever S. C. Legutko B. Garcia-Caceres C. de la Fuente E.. (2014). Hormones and diet, but not body weight, control hypothalamic microglial activity. Glia 62, 17–25. 10.1002/glia.22580 24166765 PMC4213950

[B73] Ghasemi R. Haeri A. Dargahi L. Mohamed Z. Ahmadiani A. (2013). Insulin in the brain: sources, localization and functions. Mol. Neurobiol. 47, 145–171. 10.1007/s12035-012-8339-9 22956272

[B74] Gibney S. M. McGuinness B. Prendergast C. Harkin A. Connor T. J. (2013). Poly I:C-induced activation of the immune response is accompanied by depression and anxiety-like behaviours, kynurenine pathway activation and reduced BDNF expression. Brain Behav. Immun. 28, 170–181. 10.1016/j.bbi.2012.11.010 23201589

[B75] Godbout J. P. Moreau M. Lestage J. Chen J. Sparkman N. L. O'Connor J.. (2008). Aging exacerbates depressive-like behavior in mice in response to activation of the peripheral innate immune system. Neuropsychopharmacology 33, 2341–2351. 10.1038/sj.npp.1301649 18075491 PMC2907915

[B76] Gold A. B. Herrmann N. Swardfager W. Black S. E. Aviv R. I. Tennen G.. (2011). The relationship between indoleamine 2,3-dioxygenase activity and post-stroke cognitive impairment. J. Neuroinflammation 8:17. 10.1186/1742-2094-8-17 21324164 PMC3055827

[B77] Goldbacher E. M. Bromberger J. Matthews K. A. (2009). Lifetime history of major depression predicts the development of the metabolic syndrome in middle-aged women. Psychosom. Med. 71, 266–272. 10.1097/PSY.0b013e318197a4d5 19188528 PMC2882687

[B78] Gomez-Pinilla F. Nguyen T. T. (2012). Natural mood foods: the actions of polyphenols against psychiatric and cognitive disorders. Nutr. Neurosci. 15, 127–133. 10.1179/1476830511Y.0000000035 22334236 PMC3355196

[B79] Gregor M. F. Hotamisligil G. S. (2011). Inflammatory mechanisms in obesity. Annu. Rev. Immunol. 29, 415–445. 10.1146/annurev-immunol-031210-101322 21219177

[B80] Gulaj E. Pawlak K. Bien B. Pawlak D. (2010). Kynurenine and its metabolites in Alzheimer's disease patients. Adv. Med. Sci. 55, 204–211. 10.2478/v10039-010-0023-6 20639188

[B81] Guo M. Huang T. Y. Garza J. C. Chua S. C. Lu X. Y. (2013). Selective deletion of leptin receptors in adult hippocampus induces depression-related behaviours. Int. J. Neuropsychopharmacol. 16, 857–867. 10.1017/S1461145712000703 22932068 PMC3612133

[B82] Guo M. Lu X. Y. (2014). Leptin receptor deficiency confers resistance to behavioral effects of fluoxetine and desipramine via separable substrates. Transl. Psychiatry 4, e486. 10.1038/tp.2014.126 25463972 PMC4270309

[B83] Hakeam H. A. O'Regan P. J. Salem A. M. Bamehriz F. Y. Jomaa L. F. (2009). Inhibition of C-reactive protein in morbidly obese patients after laparoscopic sleeve gastrectomy. Obes. Surg. 19, 456–460. 10.1007/s11695-008-9729-y 18841425

[B84] Hashimoto R. Mizutani M. Ohta T. Nakazawa K. Nagatsu T. (1994). Changes in plasma tetrahydrobiopterin levels of depressives in depressive and remission phases: reconfirmed by measurement with an internal standard. Neuropsychobiology 29, 57–60. 10.1159/000119064 8170526

[B85] Henry C. J. Huang Y. Wynne A. M. Godbout J. P. (2009). Peripheral lipopolysaccharide (LPS) challenge promotes microglial hyperactivity in aged mice that is associated with exaggerated induction of both pro-inflammatory IL-1beta and anti-inflammatory IL-10 cytokines. Brain Behav. Immun. 23, 309–317. 10.1016/j.bbi.2008.09.002 18814846 PMC2692986

[B86] Hoekstra R. van den Broek W. W. Fekkes D. Bruijn J. A. Mulder P. G. Pepplinkhuizen L. (2001). Effect of electroconvulsive therapy on biopterin and large neutral amino acids in severe, medication-resistant depression. Psychiatry Res. 103, 115–123. 10.1016/S0165-1781(01)00282-7 11549400

[B87] Hryhorczuk C. Sharma S. Fulton S. E. (2013). Metabolic disturbances connecting obesity and depression. Front. Neurosci. 7:177. 10.3389/fnins.2013.00177 24109426 PMC3791387

[B88] Huttunen R. Syrjanen J. (2013). Obesity and the risk and outcome of infection. Int. J. Obes. (Lond.) 37, 333–340. 10.1038/ijo.2012.62 22546772

[B89] Hwang L. L. Wang C. H. Li T. L. Chang S. D. Lin L. C. Chen C. P.. (2010). Sex differences in high-fat diet-induced obesity, metabolic alterations and learning, and synaptic plasticity deficits in mice. Obesity (Silver Spring) 18, 463–469. 10.1038/oby.2009.273 19730425

[B90] Kanasaki K. Koya D. (2011). Biology of obesity: lessons from animal models of obesity. J. Biomed. Biotechnol. 2011:197636. 10.1155/2011/197636 21274264 PMC3022217

[B91] Kanneganti T. D. Dixit V. D. (2012). Immunological complications of obesity. Nat. Immunol. 13, 707–712. 10.1038/ni.2343 22814340

[B92] Kanoski S. E. Davidson T. L. (2011). Western diet consumption and cognitive impairment: links to hippocampal dysfunction and obesity. Physiol. Behav. 103, 59–68. 10.1016/j.physbeh.2010.12.003 21167850 PMC3056912

[B93] Kariharan T. Nanayakkara G. Parameshwaran K. Bagasrawala I. Ahuja M. Abdel-Rahman E.. (2015). Central activation of PPAR-gamma ameliorates diabetes induced cognitive dysfunction and improves BDNF expression. Neurobiol. Aging 36, 1451–1461. 10.1016/j.neurobiolaging.2014.09.028 25510319

[B94] Kelley K. W. O'Connor J. C. Lawson M. A. Dantzer R. Rodriguez-Zas S. L. McCusker R. H. (2013). Aging leads to prolonged duration of inflammation-induced depression-like behavior caused by Bacillus Calmette-Guerin. Brain Behav. Immun. 32, 63–69. 10.1016/j.bbi.2013.02.003 23454036 PMC3686980

[B95] Kettenmann H. Hanisch U. K. Noda M. Verkhratsky A. (2011). Physiology of microglia. Physiol. Rev. 91, 461–553. 10.1152/physrev.00011.2010 21527731

[B96] Kim D. Kim J. Yoon J. H. Ghim J. Yea K. Song P.. (2014). CXCL12 secreted from adipose tissue recruits macrophages and induces insulin resistance in mice. Diabetologia 57, 1456–1465. 10.1007/s00125-014-3237-5 24744121

[B97] Kleinridders A. Konner A. C. Bruning J. C. (2009). CNS-targets in control of energy and glucose homeostasis. Curr. Opin. Pharmacol. 9, 794–804. 10.1016/j.coph.2009.10.006 19884043

[B98] Lafrance V. Inoue W. Kan B. Luheshi G. N. (2010). Leptin modulates cell morphology and cytokine release in microglia. Brain Behav. Immun. 24, 358–365. 10.1016/j.bbi.2009.11.003 19922787

[B99] Lasselin J. Capuron L. (2014). Chronic low-grade inflammation in metabolic disorders: relevance for behavioral symptoms. Neuroimmunomodulation 21, 95–101. 10.1159/000356535 24557041

[B100] Lasselin J. Magne E. Beau C. Ledaguenel P. Dexpert S. Aubert A.. (2013). Adipose inflammation in obesity: relationship with circulating levels of inflammatory markers and association with surgery-induced weight loss. J. Clin. Endocrinol. Metab. 99, E53–E61. 10.1210/jc.2013-2673 24243638

[B101] Lawrence C. B. Brough D. Knight E. M. (2012). Obese mice exhibit an altered behavioural and inflammatory response to lipopolysaccharide. Dis. Model. Mech. 5, 649–659. 10.1242/dmm.009068 22328591 PMC3424462

[B102] Lawson M. A. Kelley K. W. Dantzer R. (2011). Intracerebroventricular administration of HIV-1 Tat induces brain cytokine and indoleamine 2,3-dioxygenase expression: a possible mechanism for AIDS comorbid depression. Brain Behav. Immun. 25, 1569–1575. 10.1016/j.bbi.2011.05.006 21620953 PMC3191256

[B103] Lawson M. A. McCusker R. H. Kelley K. W. (2013). Interleukin-1 beta converting enzyme is necessary for development of depression-like behavior following intracerebroventricular administration of lipopolysaccharide to mice. J. Neuroinflammation 10:54. 10.1186/1742-2094-10-54 23634700 PMC3663735

[B104] Layé S. Parnet P. Goujon E. Dantzer R. (1994). Peripheral administration of lipopolysaccharide induces the expression of cytokine transcripts in the brain and pituitary of mice. Brain Res. Mol. Brain Res. 27, 157–162. 10.1016/0169-328X(94)90197-X 7877446

[B105] Ledochowski M. Murr C. Widner B. Fuchs D. (1999). Association between insulin resistance, body mass and neopterin concentrations. Clin. Chim. Acta 282, 115–123. 10.1016/S0009-8981(99)00019-4 10340439

[B106] Lehr S. Hartwig S. Sell H. (2012). Adipokines: a treasure trove for the discovery of biomarkers for metabolic disorders. Proteomics Clin. Appl. 6, 91–101. 10.1002/prca.201100052 22213627

[B107] Lepinay A. L. Larrieu T. Joffre C. Acar N. Garate I. Castanon N.. (2015). Perinatal high-fat diet increases hippocampal vulnerability to the adverse effects of subsequent high-fat feeding. Psychoneuroendocrinology 53C, 82–93. 10.1016/j.psyneuen.2014.12.008 25614359

[B108] Lestage J. Verrier D. Palin K. Dantzer R. (2002). The enzyme indoleamine 2,3-dioxygenase is induced in the mouse brain in response to peripheral administration of lipopolysaccharide and superantigen. Brain Behav. Immun. 16, 596–601. 10.1016/S0889-1591(02)00014-4 12401474

[B109] Li X. L. Aou S. Oomura Y. Hori N. Fukunaga K. Hori T. (2002). Impairment of long-term potentiation and spatial memory in leptin receptor-deficient rodents. Neuroscience 113, 607–615. 10.1016/S0306-4522(02)00162-8 12150780

[B110] Lin H. Y. Huang C. K. Tai C. M. Lin H. Y. Kao Y. H. Tsai C. C.. (2013). Psychiatric disorders of patients seeking obesity treatment. BMC Psychiatry 13:1. 10.1186/1471-244X-13-1 23281653 PMC3543713

[B111] Liu B. Kuang L. Liu J. (2014). Bariatric surgery relieves type 2 diabetes and modulates inflammatory factors and coronary endothelium eNOS/iNOS expression in db/db mice. Can. J. Physiol. Pharmacol. 92, 70–77. 10.1139/cjpp-2013-0034 24383875

[B112] Lopresti A. L. Drummond P. D. (2013). Obesity and psychiatric disorders: commonalities in dysregulated biological pathways and their implications for treatment. Prog. Neuropsychopharmacol. Biol. Psychiatry 45, 92–99. 10.1016/j.pnpbp.2013.05.005 23685202

[B113] Lu A. Steiner M. A. Whittle N. Vogl A. M. Walser S. M. Ableitner M.. (2008). Conditional mouse mutants highlight mechanisms of corticotropin-releasing hormone effects on stress-coping behavior. Mol. Psychiatry 13, 1028–1042. 10.1038/mp.2008.51 18475271

[B114] Luppino F. S. de Wit L. M. Bouvy P. F. Stijnen T. Cuijpers P. Penninx B. W.. (2010). Overweight, obesity, and depression: a systematic review and meta-analysis of longitudinal studies. Arch. Gen. Psychiatry 67, 220–229. 10.1001/archgenpsychiatry.2010.2 20194822

[B115] Manco M. Fernandez-Real J. M. Equitani F. Vendrell J. Valera Mora M. E. Nanni G.. (2007). Effect of massive weight loss on inflammatory adipocytokines and the innate immune system in morbidly obese women. J. Clin. Endocrinol. Metab. 92, 483–490. 10.1210/jc.2006-0960 17105839

[B116] Maric T. Woodside B. Luheshi G. N. (2014). The effects of dietary saturated fat on basal hypothalamic neuroinflammation in rats. Brain Behav. Immun. 36, 35–45. 10.1016/j.bbi.2013.09.011 24075847

[B117] Martinowich K. Manji H. Lu B. (2007). New insights into BDNF function in depression and anxiety. Nat. Neurosci. 10, 1089–1093. 10.1038/nn1971 17726474

[B118] McIntyre R. S. Soczynska J. K. Liauw S. S. Woldeyohannes H. O. Brietzke E. Nathanson J.. (2012). The association between childhood adversity and components of metabolic syndrome in adults with mood disorders: results from the international mood disorders collaborative project. Int. J. Psychiatry Med. 43, 165–177. 10.2190/PM.43.2.e 22849038

[B119] McNelis J. C. Olefsky J. M. (2014). Macrophages, immunity, and metabolic disease. Immunity 41, 36–48. 10.1016/j.immuni.2014.05.010 25035952

[B120] Mellor A. L. Munn D. H. (2008). Creating immune privilege: active local suppression that benefits friends, but protects foes. Nat. Rev. Immunol. 8, 74–80. 10.1038/nri2233 18064049

[B121] Molteni R. Barnard R. J. Ying Z. Roberts C. K. Gomez-Pinilla F. (2002). A high-fat, refined sugar diet reduces hippocampal brain-derived neurotrophic factor, neuronal plasticity, and learning. Neuroscience 112, 803–814. 10.1016/S0306-4522(02)00123-9 12088740

[B122] Moreau M. Andre C. O'Connor J. C. Dumich S. A. Woods J. A. Kelley K. W.. (2008). Inoculation of Bacillus Calmette-Guerin to mice induces an acute episode of sickness behavior followed by chronic depressive-like behavior. Brain Behav. Immun. 22, 1087–1095. 10.1016/j.bbi.2008.04.001 18479887 PMC2908297

[B123] Moreau M. Lestage J. Verrier D. Mormede C. Kelley K. W. Dantzer R.. (2005). Bacille Calmette-Guerin inoculation induces chronic activation of peripheral and brain indoleamine 2,3-dioxygenase in mice. J. Infect. Dis. 192, 537–544. 10.1086/431603 15995970

[B124] Moy G. A. McNay E. C. (2012). Caffeine prevents weight gain and cognitive impairment caused by a high-fat diet while elevating hippocampal BDNF. Physiol. Behav. 10.1016/j.physbeh.2012.11.008 PMC3586379 23220362

[B125] Muller M. van Raamt F. Visseren F. L. Kalmijn S. Geerlings M. I. Mali W. P.. (2010). Metabolic syndrome and cognition in patients with manifest atherosclerotic disease: the SMART study. Neuroepidemiology 34, 83–89. 10.1159/000264825 20016217

[B126] Murr C. Widner B. Wirleitner B. Fuchs D. (2002). Neopterin as a marker for immune system activation. Curr. Drug Metab. 3, 175–187. 10.2174/1389200024605082 12003349

[B127] Myint A. M. Kim Y. K. Verkerk R. Scharpe S. Steinbusch H. Leonard B. (2007). Kynurenine pathway in major depression: evidence of impaired neuroprotection. J. Affect. Disord. 98, 143–151. 10.1016/j.jad.2006.07.013 16952400

[B128] Neurauter G. Grahmann A. V. Klieber M. Zeimet A. Ledochowski M. Sperner-Unterweger B.. (2008). Serum phenylalanine concentrations in patients with ovarian carcinoma correlate with concentrations of immune activation markers and of isoprostane-8. Cancer Lett. 272, 141–147. 10.1016/j.canlet.2008.07.002 18701209

[B129] Niculescu M. D. Lupu D. S. (2009). High fat diet-induced maternal obesity alters fetal hippocampal development. Int. J. Dev. Neurosci. 27, 627–633. 10.1016/j.ijdevneu.2009.08.005 19695321 PMC2754591

[B130] O'Connor J. C. Andre C. Wang Y. Lawson M. A. Szegedi S. S. Lestage J.. (2009a). Interferon-gamma and tumor necrosis factor-alpha mediate the upregulation of indoleamine 2,3-dioxygenase and the induction of depressive-like behavior in mice in response to bacillus Calmette-Guerin. J. Neurosci. 29, 4200–4209. 10.1523/JNEUROSCI.5032-08.2009 19339614 PMC2835569

[B131] O'Connor J. C. Lawson M. A. Andre C. Briley E. M. Szegedi S. S. Lestage J.. (2009b). Induction of IDO by bacille Calmette-Guerin is responsible for development of murine depressive-like behavior. J. Immunol. 182, 3202–3212. 10.4049/jimmunol.0802722 19234218 PMC2664258

[B132] O'Connor J. C. Lawson M. A. Andre C. Moreau M. Lestage J. Castanon N.. (2009c). Lipopolysaccharide-induced depressive-like behavior is mediated by indoleamine 2,3-dioxygenase activation in mice. Mol. Psychiatry 14, 511–522. 10.1038/sj.mp.4002148 18195714 PMC2683474

[B133] Ohta R. Shigemura N. Sasamoto K. Koyano K. Ninomiya Y. (2003). Conditioned taste aversion learning in leptin-receptor-deficient db/db mice. Neurobiol. Learn. Mem. 80, 105–112. 10.1016/S1074-7427(03)00046-7 12932425

[B134] Oxenkrug G. F. (2010). Metabolic syndrome, age-associated neuroendocrine disorders, and dysregulation of tryptophan-kynurenine metabolism. Ann. N.Y. Acad. Sci. 1199, 1–14. 10.1111/j.1749-6632.2009.05356.x 20633104

[B135] Oxenkrug G. F. Requintina P. J. Mikolich D. L. Ruthazer R. Viveiros K. Lee H.. (2011). Neopterin as a marker of response to antiviral therapy in hepatitis C virus patients. Hepat. Res. Treat. 2012, 619609. 10.1155/2012/619609 22811898 PMC3395212

[B136] Page K. C. Jones E. K. Anday E. K. (2014). Maternal and postweaning high-fat diets disturb hippocampal gene expression, learning, and memory function. Am. J. Physiol. Regul. Integr. Comp. Physiol. 306, R527–R537. 10.1152/ajpregu.00319.2013 24523341

[B137] Pan A. Keum N. Okereke O. I. Sun Q. Kivimaki M. Rubin R. R.. (2012). Bidirectional association between depression and metabolic syndrome: a systematic review and meta-analysis of epidemiological studies. Diabetes Care 35, 1171–1180. 10.2337/dc11-2055 22517938 PMC3329841

[B138] Park H. S. Park J. Y. Yu R. (2005). Relationship of obesity and visceral adiposity with serum concentrations of CRP, TNF-alpha and IL-6. Diabetes Res. Clin. Pract. 69, 29–35. 10.1016/j.diabres.2004.11.007 15955385

[B139] Park S. E. Dantzer R. Kelley K. W. McCusker R. H. (2011). Central administration of insulin-like growth factor-I decreases depressive-like behavior and brain cytokine expression in mice. J. Neuroinflammation 8:12. 10.1186/1742-2094-8-12 21306618 PMC3045937

[B140] Pedersen B. K. Febbraio M. A. (2012). Muscles, exercise and obesity: skeletal muscle as a secretory organ. Nat. Rev. Endocrinol. 8, 457–465. 10.1038/nrendo.2012.49 22473333

[B141] Pistell P. J. Morrison C. D. Gupta S. Knight A. G. Keller J. N. Ingram D. K.. (2010). Cognitive impairment following high fat diet consumption is associated with brain inflammation. J. Neuroimmunol. 219, 25–32. 10.1016/j.jneuroim.2009.11.010 20004026 PMC2823983

[B142] Platten M. von Knebel Doeberitz N. Oezen I. Wick W. Ochs K. (2015). Cancer immunotherapy by targeting IDO1/TDO and their downstream effectors. Front. Immunol. 5:673. 10.3389/fimmu.2014.00673 25628622 PMC4290671

[B143] Pohl J. Woodside B. Luheshi G. N. (2009). Changes in hypothalamically mediated acute-phase inflammatory responses to lipopolysaccharide in diet-induced obese rats. Endocrinology 150, 4901–4910. 10.1210/en.2009-0526 19797120

[B144] Raison C. L. Dantzer R. Kelley K. W. Lawson M. A. Woolwine B. J. Vogt G.. (2010). CSF concentrations of brain tryptophan and kynurenines during immune stimulation with IFN-alpha: relationship to CNS immune responses and depression. Mol. Psychiatry 15, 393–403. 10.1038/mp.2009.116 19918244 PMC2844942

[B145] Raison C. L. Miller A. H. (2003). When not enough is too much: the role of insufficient glucocorticoid signaling in the pathophysiology of stress-related disorders. Am. J. Psychiatry 160, 1554–1565. 10.1176/appi.ajp.160.9.1554 12944327

[B146] Ramos-Rodriguez J. J. Molina-Gil S. Ortiz-Barajas O. Jimenez-Palomares M. Perdomo G. Cozar-Castellano I.. (2014). Central proliferation and neurogenesis is impaired in type 2 diabetes and prediabetes animal models. PLoS ONE 9:e89229. 10.1371/journal.pone.0089229 24586614 PMC3930705

[B147] Ransohoff R. M. Perry V. H. (2009). Microglial physiology: unique stimuli, specialized responses. Annu. Rev. Immunol. 27, 119–145. 10.1146/annurev.immunol.021908.132528 19302036

[B148] Rao S. R. (2012). Inflammatory markers and bariatric surgery: a meta-analysis. Inflamm. Res. 61, 789–807. 10.1007/s00011-012-0473-3 22588278

[B149] Roberts R. O. Geda Y. E. Knopman D. S. Cha R. H. Boeve B. F. Ivnik R. J.. (2010). Metabolic syndrome, inflammation, and nonamnestic mild cognitive impairment in older persons: a population-based study. Alzheimer Dis. Assoc. Disord. 24, 11–18. 10.1097/WAD.0b013e3181a4485c 19568151 PMC2837096

[B150] Rosenbaum M. Leibel R. L. (2014). 20 years of leptin: role of leptin in energy homeostasis in humans. J. Endocrinol. 223, T83–T96. 10.1530/joe-14-0358 25063755 PMC4454393

[B151] Rummel C. Inoue W. Poole S. Luheshi G. N. (2010). Leptin regulates leukocyte recruitment into the brain following systemic LPS-induced inflammation. Mol. Psychiatry 15, 523–534. 10.1038/mp.2009.98 19773811

[B152] Ryan A. S. Nicklas B. J. (2004). Reductions in plasma cytokine levels with weight loss improve insulin sensitivity in overweight and obese postmenopausal women. Diabetes Care 27, 1699–1705. 10.2337/diacare.27.7.1699 15220249

[B153] Sabia S. Kivimaki M. Shipley M. J. Marmot M. G. Singh-Manoux A. (2009). Body mass index over the adult life course and cognition in late midlife: the Whitehall II Cohort Study. Am. J. Clin. Nutr. 89, 601–607. 10.3945/ajcn.2008.26482 19073790 PMC2714395

[B154] Salazar A. Gonzalez-Rivera B. L. Redus L. Parrott J. M. O'Connor J. C. (2012). Indoleamine 2,3-dioxygenase mediates anhedonia and anxiety-like behaviors caused by peripheral lipopolysaccharide immune challenge. Horm. Behav. 62, 202–209. 10.1016/j.yhbeh.2012.03.010 22504306 PMC3425718

[B155] Savitz J. Dantzer R. Wurfel B. E. Victor T. A. Ford B. N. Bodurka J.. (2015a). Neuroprotective kynurenine metabolite indices are abnormally reduced and positively associated with hippocampal and amygdalar volume in bipolar disorder. Psychoneuroendocrinology 52, 200–211. 10.1016/j.psyneuen.2014.11.015 25486577 PMC4297593

[B156] Savitz J. Drevets W. C. Smith C. M. Victor T. A. Wurfel B. E. Bellgowan P. S.. (2015b). Putative neuroprotective and neurotoxic kynurenine pathway metabolites are associated with hippocampal and amygdalar volumes in subjects with major depressive disorder. Neuropsychopharmacology 40, 463–471. 10.1038/npp.2014.194 25074636 PMC4443961

[B157] Schmidt M. I. Duncan B. B. (2003). Diabesity: an inflammatory metabolic condition. Clin. Chem. Lab. Med. 41, 1120–1130. 10.1515/CCLM.2003.174 14598860

[B158] Schneck A. S. Iannelli A. Patouraux S. Rousseau D. Bonnafous S. Bailly-Maitre B.. (2014). Effects of sleeve gastrectomy in high fat diet-induced obese mice: respective role of reduced caloric intake, white adipose tissue inflammation and changes in adipose tissue and ectopic fat depots. Surg. Endosc. 28, 592–602. 10.1007/s00464-013-3211-1 24196540

[B159] Schwarcz R. Rassoulpour A. Wu H. Q. Medoff D. Tamminga C. A. Roberts R. C. (2001). Increased cortical kynurenate content in schizophrenia. Biol. Psychiatry 50, 521–530. 10.1016/S0006-3223(01)01078-2 11600105

[B160] Scott K. M. McGee M. A. Wells J. E. Oakley Browne M. A. (2008). Obesity and mental disorders in the adult general population. J. Psychosom. Res. 64, 97–105. 10.1016/j.jpsychores.2007.09.006 18158005

[B161] Shanley L. J. Irving A. J. Harvey J. (2001). Leptin enhances NMDA receptor function and modulates hippocampal synaptic plasticity. J. Neurosci. 21, RC186. 11734601 10.1523/JNEUROSCI.21-24-j0001.2001 PMC6763052

[B162] Sharma S. Fulton S. (2013). Diet-induced obesity promotes depressive-like behaviour that is associated with neural adaptations in brain reward circuitry. Int. J. Obes. (Lond.) 37, 382–389. 10.1038/ijo.2012.48 22508336

[B163] Siervo M. Arnold R. Wells J. C. Tagliabue A. Colantuoni A. Albanese E. (2012). Intentional weight loss in overweight and obese individuals and cognitive function: a systematic review and meta-analysis. Obes. Rev. 12, 968–983. 10.1111/j.1467-789X.2011.00903.x 21762426

[B164] Stone T. W. Forrest C. M. Stoy N. Darlington L. G. (2012). Involvement of kynurenines in Huntington's disease and stroke-induced brain damage. J. Neural Transm. 119, 261–274. 10.1007/s00702-011-0676-8 21695417

[B165] Stranahan A. M. Arumugam T. V. Cutler R. G. Lee K. Egan J. M. Mattson M. P. (2008). Diabetes impairs hippocampal function through glucocorticoid-mediated effects on new and mature neurons. Nat. Neurosci. 11, 309–317. 10.1038/nn2055 18278039 PMC2927988

[B166] Stranahan A. M. Lee K. Martin B. Maudsley S. Golden E. Cutler R. G.. (2009). Voluntary exercise and caloric restriction enhance hippocampal dendritic spine density and BDNF levels in diabetic mice. Hippocampus 19, 951–961. 10.1002/hipo.20577 19280661 PMC2755651

[B167] Tehrani A. B. Nezami B. G. Gewirtz A. Srinivasan S. (2012). Obesity and its associated disease: a role for microbiota? Neurogastroenterol. Motil. 24, 305–311. 10.1111/j.1365-2982.2012.01895.x 22339979 PMC3303978

[B168] Thaler J. P. Yi C. X. Schur E. A. Guyenet S. J. Hwang B. H. Dietrich M. O.. (2012). Obesity is associated with hypothalamic injury in rodents and humans. J. Clin. Invest. 122, 153–162. 10.1172/JCI59660 22201683 PMC3248304

[B169] Tozuka Y. Kumon M. Wada E. Onodera M. Mochizuki H. Wada K. (2010). Maternal obesity impairs hippocampal BDNF production and spatial learning performance in young mouse offspring. Neurochem. Int. 57, 235–247. 10.1016/j.neuint.2010.05.015 20538025

[B170] Tozuka Y. Wada E. Wada K. (2009). Diet-induced obesity in female mice leads to peroxidized lipid accumulations and impairment of hippocampal neurogenesis during the early life of their offspring. FASEB J. 23, 1920–1934. 10.1096/fj.08-124784 19158155

[B171] Valladolid-Acebes I. Fole A. Martin M. Morales L. Victoria Cano M. Ruiz-Gayo M.. (2013). Spatial memory impairment and changes in hippocampal morphology are triggered by high-fat diets in adolescent mice. Is there a role of leptin? Neurobiol. Learn. Mem. 106, 18–25. 10.1016/j.nlm.2013.06.012 23820496

[B172] Varga O. Harangi M. Olsson I. A. Hansen A. K. (2009). Contribution of animal models to the understanding of the metabolic syndrome: a systematic overview. Obes. Rev. 11, 792–807. 10.1111/j.1467-789X.2009.00667.x 19845867

[B173] Velloso L. A. Araujo E. P. de Souza C. T. (2008). Diet-induced inflammation of the hypothalamus in obesity. Neuroimmunomodulation 15, 189–193. 10.1159/000153423 18781083

[B174] Verdam F. J. Fuentes S. de Jonge C. Zoetendal E. G. Erbil R. Greve J. W.. (2013). Human intestinal microbiota composition is associated with local and systemic inflammation in obesity. Obesity (Silver Spring) 21, E607–E615. 10.1002/oby.20466 23526699

[B175] Volkow N. D. Wang G. J. Baler R. D. (2011). Reward, dopamine and the control of food intake: implications for obesity. Trends Cogn. Sci. 15, 37–46. 10.1016/j.tics.2010.11.001 21109477 PMC3124340

[B176] Wang Y. Lawson M. A. Dantzer R. Kelley K. W. (2009). LPS-induced indoleamine 2,3-dioxygenase is regulated in an interferon-gamma-independent manner by a JNK signaling pathway in primary murine microglia. Brain Behav. Immun. 24, 201–209. 10.1016/j.bbi.2009.06.152 19577630 PMC2818058

[B177] Wosiski-Kuhn M. Erion J. R. Gomez-Sanchez E. P. Gomez-Sanchez C. E. Stranahan A. M. (2014). Glucocorticoid receptor activation impairs hippocampal plasticity by suppressing BDNF expression in obese mice. Psychoneuroendocrinology 42, 165–177. 10.1016/j.psyneuen.2014.01.020 24636513 PMC4426342

[B178] Wynne A. M. Henry C. J. Huang Y. Cleland A. Godbout J. P. (2010). Protracted downregulation of CX3CR1 on microglia of aged mice after lipopolysaccharide challenge. Brain Behav. Immun. 24, 1190–1201. 10.1016/j.bbi.2010.05.011 20570721 PMC2939290

[B179] Xie W. Cai L. Yu Y. Gao L. Xiao L. He Q.. (2014). Activation of brain indoleamine 2,3-dioxygenase contributes to epilepsy-associated depressive-like behavior in rats with chronic temporal lobe epilepsy. J. Neuroinflammation 11:41. 10.1186/1742-2094-11-41 24594021 PMC3975854

[B180] Xu H. Barnes G. T. Yang Q. Tan G. Yang D. Chou C. J.. (2003). Chronic inflammation in fat plays a crucial role in the development of obesity-related insulin resistance. J. Clin. Invest. 112, 1821–1830. 10.1172/JCI200319451 14679177 PMC296998

[B181] Yamada K. Nabeshima T. (2003). Brain-derived neurotrophic factor/TrkB signaling in memory processes. J. Pharmacol. Sci. 91, 267–270. 10.1254/jphs.91.267 12719654

[B182] Yamada N. Katsuura G. Ochi Y. Ebihara K. Kusakabe T. Hosoda K.. (2011). Impaired CNS leptin action is implicated in depression associated with obesity. Endocrinology 152, 2634–2643. 10.1210/en.2011-0004 21521746

[B183] Yang P. J. Lee W. J. Tseng P. H. Lee P. H. Lin M. T. Yang W. S. (2014). Bariatric surgery decreased the serum level of an endotoxin-associated marker: lipopolysaccharide-binding protein. Surg. Obes. Relat. Dis. 10, 1182–1187. 10.1016/j.soard.2014.02.022 24713521

[B184] Zeyda M. Huber J. Prager G. Stulnig T. M. (2011). Inflammation correlates with markers of T-cell subsets including regulatory T cells in adipose tissue from obese patients. Obesity (Silver Spring) 19, 743–748. 10.1038/oby.2010.123 20508627

[B185] Zhang H. Wang Y. Zhang J. Potter B. J. Sowers J. R. Zhang C. (2011). Bariatric surgery reduces visceral adipose inflammation and improves endothelial function in type 2 diabetic mice. Arterioscler. Thromb. Vasc. Biol. 31, 2063–2069. 10.1161/ATVBAHA.111.225870 21680898 PMC3158262

[B186] Zhang X. Zhang G. Zhang H. Karin M. Bai H. Cai D. (2008). Hypothalamic IKKbeta/NF-kappaB and ER stress link overnutrition to energy imbalance and obesity. Cell 135, 61–73. 10.1016/j.cell.2008.07.043 18854155 PMC2586330

[B187] Zunszain P. A. Anacker C. Cattaneo A. Carvalho L. A. Pariante C. M. (2011). Glucocorticoids, cytokines and brain abnormalities in depression. Prog. Neuropsychopharmacol. Biol. Psychiatry 35, 722–729. 10.1016/j.pnpbp.2010.04.011 20406665 PMC3513408

[B188] Zunszain P. A. Anacker C. Cattaneo A. Choudhury S. Musaelyan K. Myint A. M.. (2012). Interleukin-1beta: a new regulator of the kynurenine pathway affecting human hippocampal neurogenesis. Neuropsychopharmacology 37, 939–949. 10.1038/npp.2011.277 22071871 PMC3280640

